# New generation breast cancer cell lines developed from patient-derived xenografts

**DOI:** 10.1186/s13058-020-01300-y

**Published:** 2020-06-23

**Authors:** Jessica Finlay-Schultz, Britta M. Jacobsen, Duncan Riley, Kiran V. Paul, Scott Turner, Andrea Ferreira-Gonzalez, J. Chuck Harrell, Peter Kabos, Carol A. Sartorius

**Affiliations:** 1 Department of Pathology, University of Colorado Anschutz Medical Campus, Aurora, CO 80045 USA; 2 Department of Medicine, Division of Medical Oncology, University of Colorado Anschutz Medical Campus, Aurora, CO 80045 USA; 3 Department of Pathology, Virginia Commonwealth University, Richmond, VA 23298 USA

**Keywords:** Breast cancer, Breast cancer subtypes, Breast cancer cell lines, Estrogen receptor, Patient-derived xenografts

## Abstract

**Background:**

Breast cancer is a highly heterogeneous disease characterized by multiple histologic and molecular subtypes. While a myriad of breast cancer cell lines have been developed over the past 60 years, estrogen receptor alpha (ER)+ disease and some mutations associated with this subtype remain underrepresented. Here we describe six breast cancer cell lines derived from patient-derived xenografts (PDX) and their general characteristics.

**Methods:**

Established breast cancer PDX were processed into cell suspensions and placed into standard 2D cell culture; six emerged into long-term passageable cell lines. Cell lines were assessed for protein expression of common luminal, basal, and mesenchymal markers, growth assessed in response to estrogens and endocrine therapies, and RNA-seq and oncogenomics testing performed to compare relative transcript levels and identify putative oncogenic drivers.

**Results:**

Three cell lines express ER and two are also progesterone receptor (PR) positive; PAM50 subtyping identified one line as luminal A. One of the ER+PR+ lines harbors a D538G mutation in the gene for ER (ESR1), providing a natural model that contains this endocrine-resistant genotype. The third ER+PR^−/low^ cell line has mucinous features, a rare histologic type of breast cancer. The three other lines are ER− and represent two basal-like and a mixed ductal/lobular breast cancer. The cell lines show varied responses to tamoxifen and fulvestrant, and three were demonstrated to regrow tumors in vivo. RNA sequencing confirms all cell lines are human and epithelial. Targeted oncogenomics testing confirmed the noted ESR1 mutation in addition to other mutations (i.e., PIK3CA, BRCA2, CCND1, NF1, TP53, MYC) and amplifications (i.e., FGFR1, FGFR3) frequently found in breast cancers.

**Conclusions:**

These new generation breast cancer cell lines add to the existing repository of breast cancer models, increase the number of ER+ lines, and provide a resource that can be genetically modified for studying several important clinical breast cancer features.

## Background

Breast cancer comprises a complex and diverse group of neoplasms that are stratified into various subtypes to guide treatment [1]. The three-marker diagnostic system of estrogen receptor alpha (ER), progesterone receptor (PR), and HER2 remains the cornerstone of clinical decision-making with prognostic and predictive value. In addition, the WHO classifies invasive carcinomas into a dozen more common histological subtypes, plus several rare subtypes, based on architectural and morphological features [2]. Among these, invasive ductal carcinomas (IDC) comprise the vast majority of cases (> 70%). However, it is increasingly recognized that other histological subtypes such as invasive lobular carcinomas (ILC) have unique morphologic and clinical attributes [3, 4]. Molecular classifications that emerged in the 2000s use gene expression analysis to define subtypes with different recurrence and survival patterns, for example, the PAM50 classifier [5], or a specific triple-negative breast cancer (TNBC) classifier [6]. Breast cancers can also be characterized by gene mutation status such as germline BRCA1/2 and somatic PIK3CA mutations [7–9], which can indicate eligibility for targeted inhibitors to PARP or PIK3CA, respectively, or by progressive mutation in the gene for ER (ESR1) which indicates loss of sensitivity to some endocrine therapies [10]. Existing human breast cancer cell lines, patient-derived xenografts (PDX), patient-derived organoids (PDO), and murine models with intact immune systems capture many of these unique histopathological, molecular, and genetic subtypes. Here we describe generation of six PDX-derived breast cancer cell lines including three ER+ lines, one with ESR1 mutation, that add to the collection of research models available to investigators.

In vitro propagating cell lines are the most long-standing and heavily used models for conducting basic breast cancer research. The first breast cancer cell line was generated from a primary breast IDC by Lasfargues et al. over 60 years ago named BT-20, a basal-like TNBC [11]. In the 1970s, the first ER+ lines were developed including two from late stage pleural effusions (MCF7 [12] and T47D [13]), and two from primary IDCs (BT-474, also HER2 amplified, and BT-483 [14]). Larger collections were described over a 20-year period including 19 from the MD Anderson (MDA) [15], 18 from the Hamon Cancer Center (HCC, UT Southwestern) [16], and 12 from the University of Michigan (SUM) series [17]. There are numerous other descriptions of generating single or several cell lines within focused research articles. Collectively, these endeavors have produced approximately 51 human breast cancer cell lines available for research [18]. More recently, breast cancer cell lines have been developed from circulating tumor cells [19, 20]. A survey of PubMed shows > 40,000 original research articles utilizing MCF7 cells and > 14,000 for MDA-MB-231 cells. These well-characterized cell lines are commonly manipulated to over or under express specific genes, test specific drugs, and perform genome-wide functional genetic and drug screens. This research has led to the discovery of a multitude of (mostly) cell intrinsic behaviors. Despite their historic importance, the evolving emphasis on personalized therapies necessitates continued generation of newer models that capture tumor diversity found in the population.

We previously reported the generation of a collection of PDX with an emphasis on ER+ models [21]. These provide a continuously propagating in vivo resource for testing therapeutics in a heterogeneous context, and there are > 500 such models developed by groups worldwide [22]. PDO offer an ex vivo approach to screen different patients’ tumors for drug sensitivity and can potentially include other cell types contained in the microenvironment [23]. However, PDX and PDO are time-consuming and molecular biology approaches including shRNA knockdown or CRISPR/Cas9 gene editing remain difficult in such models. We sought to generate cell lines from PDX that harbor underrepresented phenotypes and genotypes that allow basic molecular manipulations and mechanistic studies and that can be used in parallel with in vivo studies. We have created six such cell lines that capture several important clinical features and subtypes and present here their initial characterization.

## Methods

### Hormones

Hormones and drugs 17β-estradiol (E2), progesterone (P4), dihydrotestosterone (DHT), 4-hydroxy-Tamoxifen (4-OH-Tam), and ICI 118,551 (Fulvestrant (Fulv)) were purchased from Sigma-Aldrich (St. Louis, MO).

### Cell line generation

Derivation and propagation of PDX was described previously [21, 24]. Generation of cell lines was essentially as described by Jambal et al. [25]. Briefly, tumors were excised from animals, partitioned into ~ 5 mm chunks, and mechanically dispersed using a cell dissociation sieve and glass pestle (Sigma-Aldrich, St. Louis, MO). Cells were seeded into 6-well dishes, allowed to adhere and grown at 37 °C under 5% CO_2_ in Dulbecco’s modified minimum essential medium (DMEM)/F12 containing L-glutamine (365 mg/L) buffered with sodium bicarbonate (1200 mg/L) and HEPES (3575 mg/L) with 10% fetal bovine serum, cholera toxin (100 ng/mL), hydrocortisone (1 μg/mL), insulin (10^−9^ M), and pen strep. Cells were fed every 3–4 days until they emerged from crisis into passageable cell lines (6–18 months). Control breast cancer cell lines T47D and MDA-MB-468 were obtained from the University of Colorado Cancer Center Tissue Culture core. Short tandem repeat (STR) profiling and analysis was performed by the University of Arizona Genetics Core (University of Arizona, Tucson, AZ). PDX-derived cell lines were compared to their PDX of origin and other cell lines in the database. Cell lines were routinely tested for mycoplasma using the MycoAlert detection kit (Lonza, Basel, Switzerland).

### Immunochemistry and microscopy

For immunocytochemistry (ICC), 1–2 × 10^5^ cells were seeded onto glass coverslips in 6-well plates in regular media. For assessing PR, cells were treated with 10 nM E2 for 48 h prior to collection. Cells were washed twice with PBS and fixed with ice-cold 70% acetone/30% methanol for 5 min. Fixed cells were blocked with 10% normal goat serum (Vector Labs, Burlingame, CA) in 0.05% TBS-T for 30 min. Primary antibodies were as follows: ERα (SP1, 1:200, Thermo-Fisher, Waltham, MA), PR (1294, 1:50, [26], CK5 (ab75869, 1:400, Abcam, Cambridge, MA), CK8/18 (NCL-L-5D3, 1:2000, Leica Biosystems), and Vimentin (5741, 1100, Cell Signaling, Danvers, MA) for 1 h. Secondary antibodies were A11029 (green) and/or A11037 (red) (Invitrogen, 1:200) for 30 min followed by counterstaining with 0.1 μg/mL DAPI. Phase contrast images were captured using a Nikon TiE microscope (Nikon, Melville, NY) equipped with a digital camera and NIS Elements 4.6 software. Fluorescent images were captured using an Olympus BX40 microscope equipped with a digital camera and cellSens Standard 1.13 software. Adobe Photoshop CC 2019 was used to perform minimal linear adjustments to brightness/contrast and to assemble pictures into multipanel figures. Immunohistochemistry (IHC) of PDX was performed with the same antibodies for the indicated markers as previously described [21].

### Immunoblotting

Cells were treated with vehicle (ethanol), 10 nM E2, 100 nM P4, E2 plus P4, or 10 nM DHT for 48 h. Cell lysates were harvested in lysis buffer (50 mM Tris pH 7.4, 140 nM NaCl, 2 mM EGTA, 1% Tween-20) plus protease/phosphatase inhibitors. Primary antibodies were as follows: ERα (6F11, Santa Cruz Biotechnology (sc-56,836), 1:1000), PR (1294, 1:500), AR (441, 11,000, DAKO-Agilent, Santa Clara, CA), and α-tubulin (ST1568, 1:30,000, Sigma-Aldrich). Secondary antibodies were as follows: IRDye 800CW Goat-Anti-Mouse IgG and IRDye 680RD Goat-Anti-Rabbit IgG (all from Li-Cor Biosciences, Lincoln, NE). The Odyssey CLx Infrared Imaging System and Image Studio 5.2 (Li-Cor Biosciences) were used to capture images.

### Fluorescent tagging of cell lines and proliferation assay

Lentiviral particles were produced by co-transfecting HEK293FT cells with the structural plasmids VSV-G, d8.9, and pLJMGFP-GFP-3xNLS-puromycin constructs using TransIT-LT1. Media containing viral particles was harvested after 48 h. UCD cell lines were plated at ∼ 60,000 cells per 6 cm dish in 5 mL of complete medium. Approximately 24 h post plating, the medium was replaced with medium containing lentiviral particles. Polybrene was added at a final concentration of 8 mg/mL. Cells were transduced for 48 h, medium replaced with fresh complete medium and allowed to recover for 24 h, and selection performed over 7–10 days.

Real-time imaging (IncuCyte, Sartorius, Ann Arbor, MI) was used to measure proliferation of nuclear-GFP labeled cells at × 10 magnification. UCD4 cells were plated at 15,000 cells/well, UCD65 at 20,000 cells/well, and UCD12, UCD46, UCD115, and UCD178 cells at 10,000 cells/well, all in 96-well plates. For treatments, cells were given vehicle, 10 nM E2, 100 nM 4-OH-Tam, or 10 nM Fulv on day one. Green count was taken immediately after treatment and subsequently taken every 4 h for 6 days. Counts were calculated as fold change. Significance was assessed by one-way ANOVA/Tukey at the final time point.

### Growth of cell lines in vivo

Animal experiments were performed under an approved University of Colorado Institutional Animal Care and Use Committee protocol. Tumor xenografts were developed by injecting 1 × 10^6^ cells in 90% Cultrex Basement Membrane Extract (R&D Systems, Minneapolis, MN) into the fourth mammary fad pad of ovariectomized female NOD-scid IL2Rgamma^null^ (NSG). Silastic pellets containing 17β-estradiol (1 mg) were implanted subcutaneously at time of tumor cell injection. Tumors were measured weekly using a digital caliper, and tumor volume estimated using the formula (lw^2^)/2.

### RNA sequencing

Cell lines were plated in 60 mm plates in regular media. When cells were ~ 70% confluent, cells were washed with PBS and lysed with 0.7 mL Qiazol (Qiagen, Germantown, MD) immediately. RNA was prepared using miRNeasy mini columns (Qiagen) and treated with RNase-free DNase. RNA concentration was measured using a Nanodrop 2000 (Thermo-Fisher), and integrity analyzed using an Agilent 2100 Bioanalyzer and the RNA 6000 Nano kit. Libraries were prepared using the Illumina TruSeq Stranded mRNA Library Prep kit and samples sequenced using the Illumina Novaseq System. Paired-end 150 nt reads were aligned to the human genome version GRCh38.p13 using STAR 2.6. The downstream expression analysis was done using Cufflinks 2.2.1. PAM50 scores were assigned from the expression values (TPM) using the R package genefu 2.18.1 [27].

### Targeted oncogenic driver analysis

Samples of each of the six cell lines were submitted to the Virginia Commonwealth University Molecular Diagnostics Laboratory (MDL) for testing using the Oncomine Comprehensive Assay v3 panel (Thermo-Fisher). This assay uses an NGS platform to detect relevant SNVs, CNVs, gene fusions, and indels from 161 unique cancer driver genes (https://assets.thermofisher.com/TFS-Assets/LSG/brochures/oncomine-comprehensive-assay-v3-flyer.pdf). Data were annotated by the MDL according to the ASCO/AMP guidelines. All samples were confirmed to be human in origin.

## Results

### Generation of six breast cancer cell lines from PDX that span diverse phenotypes

Given the relative dearth of ER+PR+ cell lines in proportion to the > 75% of patients with this diagnosis, one of our primary goals was to focus on this subtype for generating long-term cultures. In addition, we attempted generation of HER2 overexpressing and TNBC cell lines. Thirteen PDX were processed for cell line generation, with 6 amenable to long-term 2D culture. There was no identifiable feature that predicted culture success, with individual ER+, HER2 amplified/ER−, and TNBC PDX failing to produce stable culture lines after multiple attempts. The time from initiation of cell culture to becoming established cell lines ranged from 6 to 18 months. Cells underwent a minimum of 20 passages prior to cell line declaration. General features of the six cell lines are described in Table 1. The PDX from which cell lines were derived fall into two general groups: an ER+ group of three PDX (UCD4, UCD12, UCD65) that have relatively high ER expression (> 50% ER+ cells), and a group of three PDX (UCD46, UCD115, UCD178) that have < 1% ER+ cells and would for the purposes of treatment be considered ER−. All are from female patients between 36 and 68 years old. Five were originally classified at time of diagnosis as IDC, one with mucinous features, and one as ILC. Three of the PDX-derived cell lines are developed from metastases and three from primary tumors.

**Table 1:** UCD PDX-derived cell line general characteristics

Cell line	Origin	Patient age	Pathology of patient specimen	PDX ER/PR status^**a**^	Cell line ER/PR status^**a**^	Treatment
UCD4	Pleura	68	Invasive mammary carcinoma. Metastatic adenocarcinoma. Grade 2. Original pathology^b^ ER+PR+Her2−	ER+++PR+++	ER+++PR+	Tamoxifen, AI, fulvestrant, chemotherapy
UCD12	Primary tumor	54	IDC with mucinous features, grade 3, ER+(93%)PR+(15%)Her2−	ER++PR−	ER++PR−	None
UCD65	Lymph node	47	Metastatic carcinoma in LN. ER(97%)PR(5%)Her2−	ER+++PR++	ER+++PR++	Neoadjuvant chemotherapy
UCD46	Primary tumor	41	IDC multifocal. Grade 3. ER(0%)PR(20%)Her2−	ER^low^PR−	ER−PR−	Neoadjuvant chemotherapy
UCD115	Primary tumor	36	IDC. ER−PR−Her2−	ER^low^PR−	ER−PR−	Neoadjuvant chemotherapy
UCD178	Pleura	ND	ILC. Original pathology^b^ ER+PR+Her2−	ER^low^PR−	ER−PR−	Tamoxifen, AI, palbociclib, chemotherapy

^a^Percent positive cells: +++, > 60%; ++, 31–60%; +, 1–30%; low, < 1%

^b^For primary tumor at diagnosis, the two pleural effusions were not re-evaluated upon recurrence

### Expression of hormone receptors and cytokeratins in new breast cancer cell lines

**Fig. 1:**
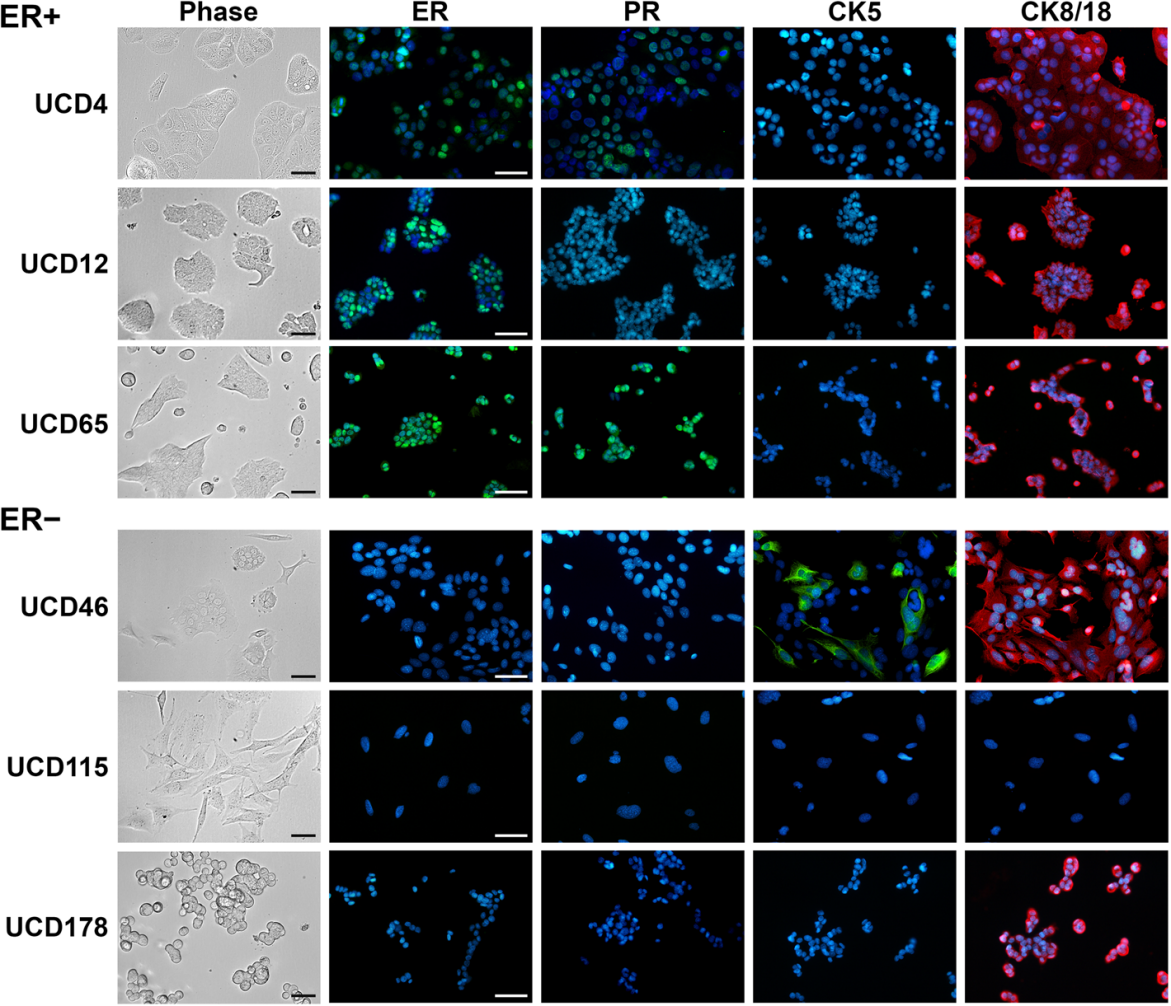
Cell morphology and expression of hormone receptors and cytokeratins in UCD breast cancer cell lines. Cells were plated on glass slides in regular media. Phase contrast images were taken on live cells. Cells were fixed and ICC performed with antibodies to ERα, PR, CK5, or CK8/18 with DAPI counterstain. For measuring PR, cells were treated with 10 nM E2 for 48 h prior to staining. Scale bars, 50 um

General cell morphology was assessed by phase contrast microscopy and is illustrated in Fig. 1. In addition, ER, PR, and cytokeratins (CK) 8/18 and CK5 as general indicators of luminal vs. basal-like epithelial cells, respectively, were measured by ICC in cells grown in basal media. For comparison, IHC for the same markers in the originating PDX is depicted in Supplemental Figure 1. The three ER+ cell lines (UCD4, UCD12, and UCD65) all grow as monolayers of cohesive cells and contain a relatively high percent of ER+ cells (> 50%), similar to their PDX of origin. For this group, PR expression was measured after 24 h incubation with 10 nM E2. E2 efficiently induced PR+ cells in UCD4 and UCD65, but not UCD12 cells. UCD4, UCD12, and UCD65 were all ubiquitously CK8/18 positive but contained no detectable CK5+ cells. In the three cell lines derived from ER−/low PDX (UCD46, UCD115, and UCD178), no ER+ or PR+ cells were detected by ICC. UCD46 cells grow in colonies and are ubiquitous for CK8/18, and most cells also express CK5, analogous to its parent PDX. UCD115 is peculiar in that cells have a more mesenchymal morphology relative to the other cell lines and do not express CK8/18 and CK5, unlike its parent PDX which is positive for both markers. UCD178 forms monolayers of rounded cells that are CK8/18+ but CK5−. Overall, with the exception of CK expression in UCD115, cell lines generally reflected their PDX of origin with regard to the 4 markers assessed. To ensure cell lines were derived from their originating PDX, cells and PDX fragments were analyzed by STR profiling. All cell lines matched their cognate PDX of origin above the 80% threshold and did not match other cell lines within the DSMZ reference database. This baseline assessment can be used to authenticate cell lines in future passages.

**Fig. 2:**
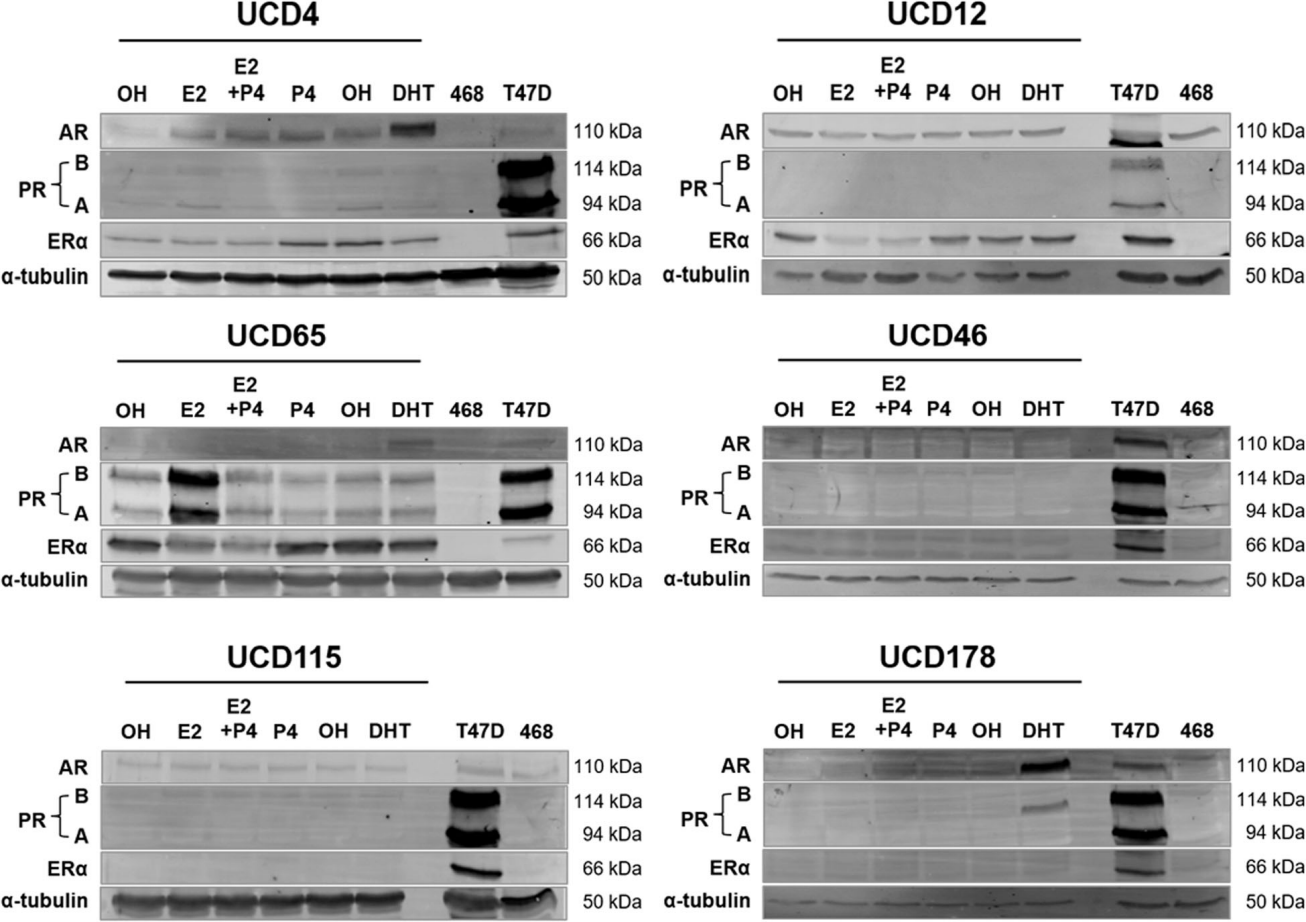
Hormone receptor expression in UCD cell lines under different hormone treatments. UCD cells were treated with EtOH (vehicle), 10 nM E2, 100 nM P4, E2+P4, or 10 nM DHT for 48 h. AR, PR, and ERα were measured by immunoblot; α-tubulin was used as a loading control. Untreated T47D and MDA-MB-468 (468) cells are shown as positive and negative controls for hormone receptor expression, respectively

Since hormone treatments can influence receptor levels through direct transcript regulation or ligand-dependent downregulation or stabilization, we additionally assessed expression of steroid receptors ER, PR, and androgen receptor (AR) by immunoblot in the presence of vehicle, E2, P4, E2+P4, or DHT (Fig. 2). T47D cells were used as positive controls and MDA-MB-468 cells as negative controls, for ER, PR, and AR. Among the three ER+ cell lines, UCD65 has the highest ER protein levels. E2 treatment caused downregulation of ER in UCD12 and UCD65, but not UCD4 cells, which contains the D538G ESR1 mutation. PR is present in UCD4 and UCD65 in the absence of hormones and is increased by E2 in UCD4 (slightly) and UCD65 cells. UCD46, UCD115, and UCD178 were ER and PR negative by immunoblot. AR was present in all three ER+ cell lines and UCD178 cells and was increased by DHT treatment. UCD46 was AR− and UCD115 has very low basal AR expression that is unaffected by DHT. Collectively, these results confirm that PDX-derived cell lines generally reflect the hormone receptor status of the originating PDX (Supplemental Figure 1). Furthermore, hormone treatments induce similar hormone receptor dynamics as observed in previously established cell lines, such as ER downregulation and PR upregulation with estrogens and AR stabilization with androgens.

### Growth of breast cancer cell lines with estrogens and endocrine therapies

**Fig. 3:**
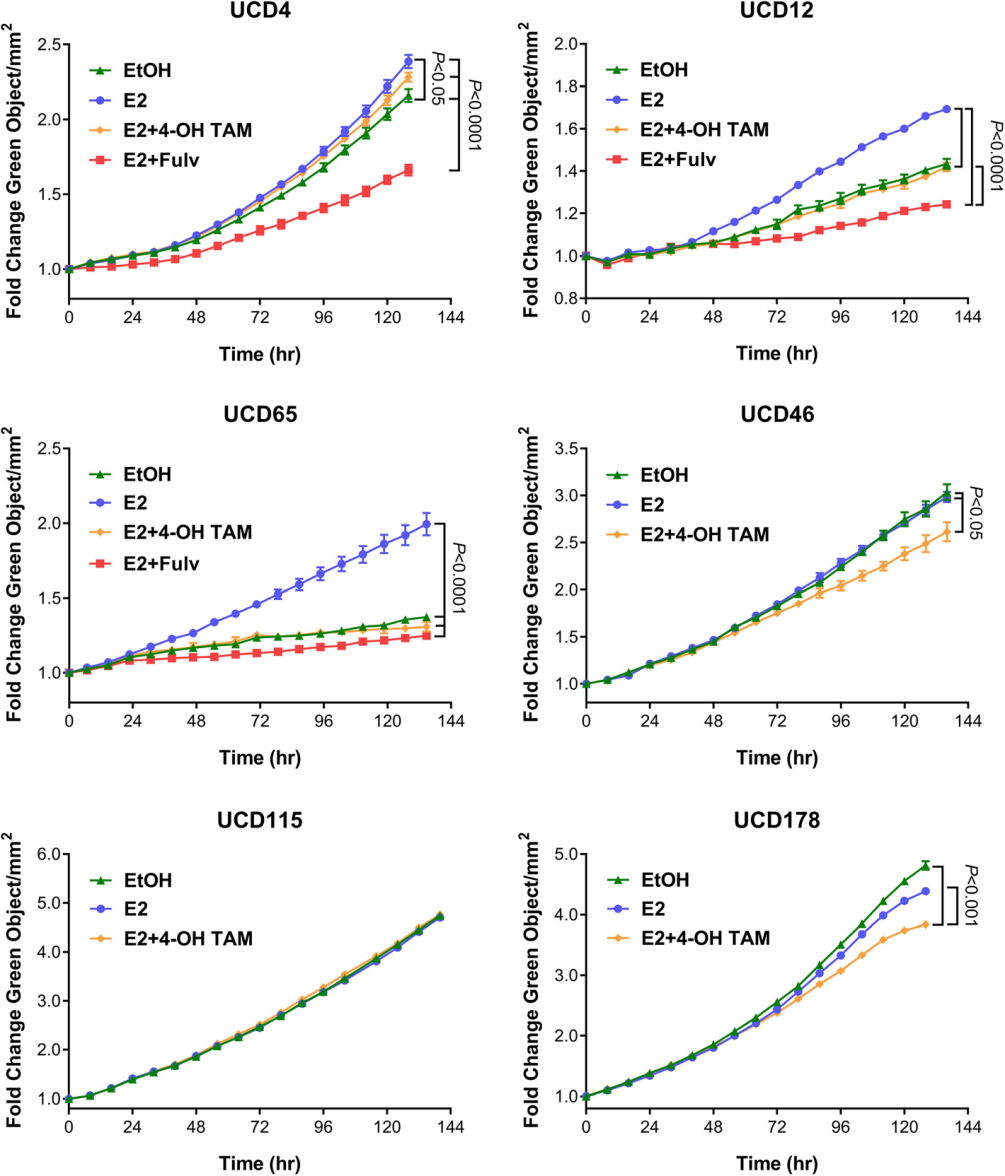
UCD cell line growth profiles under different endocrine treatments. Cell lines were placed in phenol red free media with charcoal stripped serum 24 h prior to treatment with EtOH (vehicle) or 10 nM E2. UCD4, UCD12, and UCD65 cells were additionally treated with 100 nM 4-OH TAM or 10 nM Fulv. Proliferation was measured using the IncuCyte live cell imaging system. Fold change was calculated vs. time zero; error bars show SEM. One way ANOVA/Tukey P values

Growth of PDX UCD4, UCD12, and UCD65 has been previously described [21, 24]. UCD12 and UCD65 require estrogen supplementation for in vivo growth whereas UCD4 grows tumors in non-estrogen-supplemented mice, and their growth is accelerated in the presence of estrogen. To measure the proliferation rates of the PDX-derived cell lines, we first transduced them with a nuclear-GFP construct (Supplemental Figure 2) and then measured cell number using the IncuCyte live cell imaging platform under the following conditions: vehicle, E2, and E2+4-OH-Tam, and E2 plus Fulv (ER+ cell lines only) (Fig. 3). Fold change growth from vehicle to E2 was 1.1, 1.2, and 1.6-fold for UCD4, UCD12, and UCD65, respectively. E2-induced growth was significantly attenuated by 4-OH-Tam or Fulv in UCD12 and UCD65 and by Fulv only in UCD4.

Growth of all three ER− lines (UCD46, UCD115, UCD178) was unaffected by E2 treatment. However, UCD46 and UCD178 showed a very modest reduction in growth in the presence of E2+4-OH-Tam vs vehicle or E2 alone. Cell population doubling times in the absence and presence of E2 are summarized in Table 2. All new cell lines were notably slower growing than T47D or MDA-MB-468 cells, a phenomenon previously observed for newly cultured breast cancer cells [17]. Baseline doubling times were slower on average for ER+ vs. ER− cell lines, with E2 decreasing the doubling times for UCD4, UCD12, and UCD65.

**Table 2:** UCD cell line population doubling times

Cell line	Population doubling time (h)^a^	
	Vehicle	E2
T47D	59.7	33.8
MDA-MB-468	40.6	ND
UCD4	95.9	89.3
UCD12	110.1	94.6
UCD65	132.8	109.3
UCD46	81.5	89.4
UCD115	65.4	67.7
UCD178	54.1	55.8

^a^Calculated using the formula PDT = time(h)*log (2)/(log (average cell count at 96 h)-log (average cell count at 48 h))

To test if cell lines are tumorigenic in vivo, we inoculated UCD12, UCD46, and UCD65 cells into the #4 mammary fat pads of female NSG mice supplemented with estrogen (Supplemental Figure 3). All three cell lines grew into solid tumors in response to estrogen. In addition, the UCD12 cell line was previously demonstrated to be tumorigenic in vivo [28].

### Genomic and transcriptomic characterization of cell lines

**Fig. 4:**
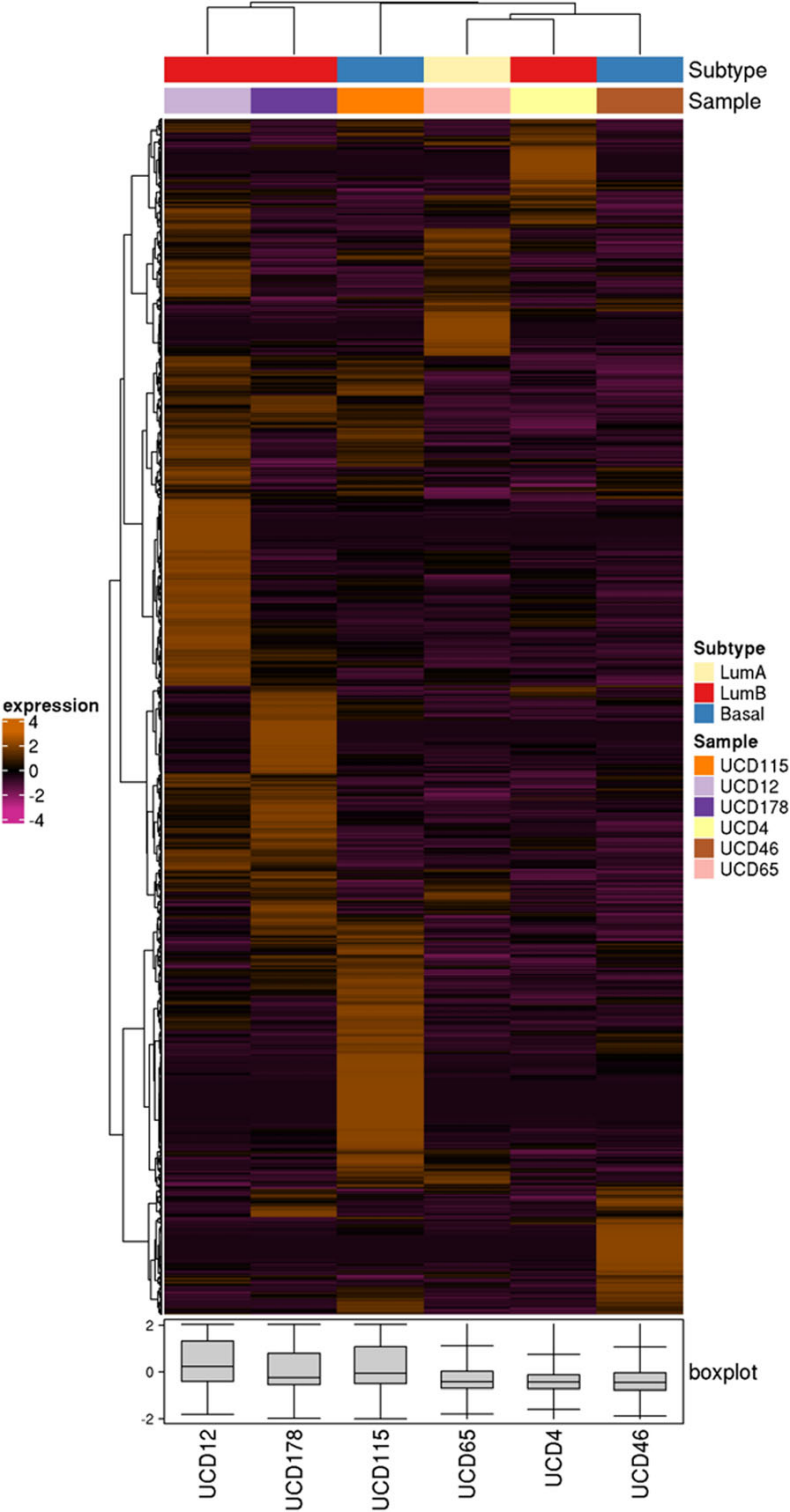
PAM50 intrinsic molecular subtypes of UCD breast cancer cell lines. PAM50 scores were assigned using the bioclassifier R code described by Gendoo et al. [27] using RNA-seq data for the six cell lines and a panel of 18 UCD PDX of varied subtypes

To measure and compare gene expression patterns across the six cell lines, RNA-seq was performed on cells grown in regular media in the absence of hormone supplementation. Normalized expression of transcripts is summarized in Supplemental Table 1. All cell lines aligned to the human genome. The expression profiles of UCD cell lines were compared to their PDX of origin and show moderate to strong positive correlation coefficients (Supplementary Figure 4). The relative transcript level for individual genes can be assessed and compared, with levels of ESR1, for example, generally matching the ER protein levels in depicted in Figs. 1 and 2. Likewise, transcript levels for PR (*PGR*), AR, CK5 (*KRT5*), and CK8/18 (*KRT8/KRT18*) are generally reflective of their protein levels. By RNA-seq, UCD115 has comparatively high expression of vimentin (*VIM*), N-cadherin (*CDH2*), and Slug (*SNAIL2*), for example, and low expression of all cytokeratin genes (i.e., *KRT18*) and E-cadherin (*CDH1*). The three ER− cell lines all contain vimentin-positive cells (Supplemental Figure 5). None of the cell lines overexpressed HER2 transcripts (*ERBB2*), consistent with the original tumor diagnosis. All cell lines were absent for CD45 (*PTPRC*), a marker of hematopoietic cell lineage, suggesting none of the cell lines were derived from lymphoma. We next applied the intrinsic molecular subtype algorithm described by Parker et al. [29] to the cell line RNA-seq data. This analysis identified the UCD65 cell line as luminal A, UCD4, UCD12 and UCD178 as luminal B, and UCD46 and UCD115 as basal-like breast cancer subtypes (Fig. 4).

To assess common oncogenic alterations in the six cell lines, we utilized a targeted genomic testing platform that covers SNVs, CNVs, and gene fusions for 161 cancer driver genes (Table 3). All of the cell lines contained somatic mutations and amplifications commonly described in breast cancer [9]. Mutations in the ER+ cell lines include BRCA2, ESR1 (D538G, confirming the Sanger sequencing), PIK3CA, and NF1. All three ER+ cell lines had amplification of FGF receptors (FGFR1 and/or FGFR3) and/or FGF ligands (FGF3, FGF19) and one has amplification of cyclin D (CCND1). Within the ER− cell lines, mutations were found in TP53, PIK3CA, and NF1. UCD115 and UCD178 have amplification of MYC, UCD46 has amplification of PIK3CA and Cyclin E (CCND2), and UCD178 has a SEC16A-NOTCH1 fusion. Taken together, each cell line harbors commonly described driver mutations for breast cancers with notable events typical for ER+ and TNBCs present in each cell line.

**Table 3:** UCD cell line oncogenic alterations as determined by oncomine comprehensive assay v3

Cell line	Gene	Chromosome	Protein	Class	AF/CN
UCD4	BRCA2	c.1755_1759delGAAAA	p.K585fs*3	P	98%
	ESR1	c.1613A>G	D538G	P	100%
	FGFR3	Amplification		P	4.63 copies
	RAF1	Amplification		LP	4.91 copies
	RAD51B	c.246C>G	p.F82L	VUS	47%
	FANCD2	Amplification		VUS	4.63 copies
UCD12	BRCA2	c.4943delC	p.A1648fs*22	P	100%
	PIK3CA	c.3140A>T	p.H1047L	P	52%
	FGFR1	Amplification		P	12.47 copies
	RAD51C	Amplification		LP	4.69 copies
	FLT3	c.2890G>T	p.E964*	VUS	100%
	RNF43	Amplification		VUS	4.83 copies
	SLX4	c.2854_2855delGCinsAT	p.A952M	VUS	49%
	SPOP	Amplification		VUS	4.1 copies
UCD65	NF1	c.2372dupT	p.L792fs*2	LP	43%
	NOTCH2	c.6403_6404delCT	p.L2135fs*2	LP	49%
	FGFR1	Amplification		P	13.85 copies
	FGF3	Amplification		LP	9.53 copies
	FGF19	Amplification		LP	8.24 copies
	CCND1	Amplification		P	7.32 copies
	GNAS	Amplification		LP	5.19 copies
	CREBBP	c.3985C>T	p.L1329L	VUS	50%
UCD46	CCND2	Amplification		P	5.01 copies
	PIK3CA	Amplification		P	4.43 copies
	TP53	c.281C>G	p.S94*	P	100%
	CREBBP	Amplification		LP	4.22 copies
	SLX4	Amplification		VUS	4.22 copies
	TSC2	Amplification		VUS	4.29 copies
UCD115	TP53	c.892_911dupGAGCTG CCCCCAGGGAGCAC	p.K305fs*47	P	77%
	MYC	Amplification	–	P	6.75 copies
	ROS1	Amplification	–	LP	4.36 copies
	ERCC2	Amplification	–	LP	4.52 copies
	NF1	c.5399_5404del insCCCAGC	p.V1800_S1802del insAQP	VUS	49%
	PIK3CA	c.1663A>G	p.R555G	VUS	12%
	SETD2	c.3224A>T	p.N1075I	VUS	49%
UCD178	MYC	Amplification		P	6.47 copies
	MDM2	Amplification		P	5.22 copies
	RAF1	amplification		P	4.76 copies
	SEC16A-NOTCH1	Fusion		LP	
	FGFR4	c.826G>A	p.D276N	VUS	46%
	NF1	c.3976 T>A	p.L1326I	VUS	100%

*P* pathogenic, *LP* likely pathogenic, *VUS* variant of uncertain significance, *AF* allele frequency, *CN* copy number

## Discussion

The innate intertumoral heterogeneity among breast cancers and an increasing emphasis on individualizing therapies necessitate that we continue to generate research models to meet this challenge. Advances in measuring CTCs and circulating tumor DNA further facilitate real-time monitoring of disease progression and personalized care. Our group and others have derived collections of breast cancer PDX that can be utilized for pre-clinical drug testing [22]. However, some fundamental research questions still require novel human disease models that can be more feasibly engineered. Here we describe the generation of six PDX-derived passageable breast cancer cell lines that are amenable to manipulations such as viral transduction. These complement existing models, with well-annotated oncogenic driver mutations and expression profiles, to provide depth in conducting basic and translational research on breast cancer.

One of our primary goals was to increase the number of workable ER+ breast cancer cell lines, which are relatively underrepresented compared to their clinical predominance. The primary “workhorse” ER+ breast cancer models include IDC subtypes (MCF7, T47D, ZR75-1, and the ER+HER2+/amplified BT474) with several ER+ ILC cell lines seeing increased use (MDA-MB-134, MDA-MB-330, SUM44, and BCK4) [25, 30]. Several additional cell lines are reported to have ERα mRNA transcripts [18]; however, ER protein expression has not always been documented. PR is expressed only in UCD4 and UCD65; UCD65 cells have some constitutive expression of PR in the absence of estrogen likely due to their naturally high ER level. AR is present in all three cell lines to some degree but is highest in UCD4 cells where it is stabilized with DHT. A drawback of these ER+ cell lines is their relatively long doubling times compared to long-term cultured ER+ cell lines. UCD65 has the longest doubling time, which is typical of the slower growing luminal A subtype breast cancers. A slow proliferation rate is also typical of newly developed breast cancer cell lines [17] and may more accurately reflect growth rates in ER+ patients.

It is now recognized that up to 30% of advanced breast cancer patients contain somatic genetic anomalies in the ER gene (ESR1), prospectively driven by long-term estrogen deprivation with aromatase inhibitors (AIs) [31]. Existing breast cancer cell lines do not harbor ESR1 mutations naturally (cbioportal.org), even though some were derived from metastatic patients, prospectively because these patients were either untreated or treated prior to standard use of AIs in the 2000s. To functionally study the mutant ERs, laboratories have used exogenous expression or generated CRISPR knock-in models of the D538G and Y537S ESR1 mutations, or forced mutations by long-term endocrine treatment of ER+ breast cancer cells [31–34]. Some PDX models contain ESR1 mutations, notably in the WHIM collection [35]. To our knowledge, a cell line has not been derived from a specimen with a natural ESR1 mutation, without the potential unintended off-target effects of CRISPR-derived clonal cell lines, and that likely contain other anomalies that co-occur with ESR1 mutation. The UCD4 cell line is homozygous for the ESR1 D538G mutation and is Fulv but minimally Tam responsive (Fig. 3). UCD65 cells are the first cell line, to our knowledge, that is luminal A and expresses high levels of ER comparatively to the other cell lines. UCD12 (formerly PT12 cells) are ER+ have been used to study mucin secretion dynamics, anti-AR therapies, and endocrine resistance in diet-induced obesity models of breast cancer [28, 36, 37]. UCD4 and UCD12 contain BRCA2 mutations that are likely somatic although the germline status of the patients is unknown. All three cell lines contain amplification of the FGF-FGFR signaling axis including FGFR1 (UCD12 and UCD65), FGFR3 (UCD4), and FGF3 and FGF19 in UCD65. This underscores the significance of FGF signaling as driving ER+ breast cancers, and here we note these anomalies in early (primary tumor, UCD12), intermediate (lymph node, UCD65), and advanced (pleural effusion, UCD4) disease settings. Thus, the ER+ cell lines provide additional platforms for testing FGFR inhibitors [38].

The three ER−PR− cell lines (by our limits of detection) were derived from PDX which each contained rare ER+ cells (Supplemental Figure 1). However, the patient tumors were defined as ER−PR+ (UCD46), ER−PR− (UCD115), and ER+ at original diagnosis but untested in the recurrent pleural effusion (UCD178). Total loss of ER upon tumor progression occurs in only 10–20% of patients [39], and we speculate this could be the case for UCD178. We have observed that most TNBC specimens contain rare ER+ cells as PDX, prospectively due to estrogen used at initial implantation or a change in microenvironment. We speculate that the predominant ER− populations were likely selected for in culture. All three ER− cell lines also have alterations associated with impaired P53 function, either mutation in the TP53 gene (UCD115, UCD178) or amplification of its negative regulator MDM2 (UCD46). Basal-like UCD46 cells additionally have amplification of cyclin E (CCND2), PIK3CA, and CREBBP. UCD115 is peculiar as its parent PDX is epithelial-like with CK5+ and CK18+ cells, whereas the cells that grew in culture may have undergone a partial epithelial-mesenchymal transition (EMT), evidenced by loss of CKs, gain of vimentin, and increase in transcripts for EMT transcription factors (Fig. 1, Supplemental Figure 5, Supplementary Table 1). The UCD178 line was derived from a patient with ILC at the time of recurrence in the lungs (pleural effusion). Although this cell line aligned with the luminal B molecular subtype, it has low ER transcript expression and lack of ER protein. RNA-seq data for UCD178 shows expression of both E-cadherin and N-cadherin transcripts, which we confirmed by ICC (not shown). We presently describe the UCD178 cell line as mixed ductal/lobular, although further characterization is needed to define their unusual histological type. UCD178 also contains a SEC16A-NOTCH1 fusion protein occasionally found in breast cancers [40]. Thus, the three ER− cell lines share some common features of TNBC cell lines (i.e., mutation in the P53 signaling axis, MYC amplification) and harbor some less common mutations (i.e., SEC16A-NOTCH fusion) and histological features (i.e., ILC/IDC subtype).

## Conclusions

In this manuscript, we describe six PDX-derived breast cancer cell lines that can be used in parallel with existing cell lines to provide depth and rigor to experimental approaches. These include three new ER+ cell lines, including two that are ER+PR+ and one with a mixed ductal/lobular phenotype. While each of these cell lines grows comparatively slower than long-established ER+ and ER− cell lines, this may be more reflective of their true proliferative rates, and they remain amenable to serial passaging and manipulations such as viral transduction. The expanding repertoire of cells that represent the individualistic nature of breast cancer will bring us closer to realizing prevention and cure for a wider group of patients.

## Supplementary information

**Additional file : Supplemental Figure 1**. IHC of PDX from which cell lines were derived. PDX were stained with antibodies to ER, PR, AR, CK5, and CK8/18. Slides were loaded into Aperio digital slide viewer and images captured at 40x magnification with Imagescope (Leica). **Supplemental Figure 2**. Nuclear-GFP labeled UCD cell lines for proliferation assays. Representative images of UCD cell lines during Incucyte analysis. Individual wells were captured at 6-18 h post plating. Mag bars,300 microns. **Supplemental Fig. 3**. Growth of UCD cell lines in vivo. One million cells were inoculated billaterally into the #4 mammary fat pads of NSG mice. Animals were supplemented with slow release estrogen pellets and tumors measure weekly. Mean plus SEM are indicated. *N*=6 tumors UCD12 and UCD47 and *N* = 3 tumors forUCD65. **Supplemental Figure 4**. Expressison profiles of UCD cell lines show moderate to strong positive correlation to their PDX tumors of origin. The RNA-seq expression profiles (FPKM) of UCD cell lines and UCD PDX were compared using the r package ggpubr. The r2 correlation and *p* values were plotted for each cell line and thecorresponding PDX. **Supplementary Figure 5**. Vimentin expression in ER negative UCD cell lines. UCD46, UCD115, and UCD178 cells were plated on glass slides in regular media. Cells were fixed and stained by immunocytochemistry with an antibody to vimentin with DAPI counterstain. Scale bar, 50 μM.

**Additional file 2: Supplementary Table 1**. RNA-seq of UCD cell lines.

## Data Availability

The raw RNA-sequencing data for the six breast cancer cell lines is available in the NCBI Gene Expression Omnibus (GEO Accession #GSE146024). The unique cell lines are available to qualified individuals for research purposes through formal request, MTA agreement, and appropriate fee.

## Abbreviations

4-OH-Tam: 4-hydroxy-Tamoxifen
AIs: Aromatase inhibitor
AR: Androgen receptor
BRCA1/2: BReast CAncer genes 1 and 2
CCND1: Cyclin D
CCND2: Cyclin E
CK: Cytokeratin
DHT: Dihydrotestosterone
E2: 17β-estradiol
EMT: Epithelial-mesenchymal transition
ER: Estrogen receptor alpha
ESR1: Estrogen receptor alpha gene
FGF: Fibroblast growth factor
FGFR: Fibroblast growth factor receptor
Fulv: Fulvestrant, ICI 118,551
IDC: Invasive ductal carcinoma
ILC: Invasive lobular carcinoma
NF1: Nuclear factor 1
P4: Progesterone
PARP: Poly-ADP ribose polymerase
PDO: Patient-derived organoid
PDX: Patient-derived xenograft
PIK3CA: Phosphatidylinositol-4,5-bisphosphate 3-kinase catalytic subunit alpha
PR: Progesterone receptor
TNBC: Triple-negative breast cancer
