# Supplementary material for: New generation breast cancer cell lines developed from patient-derived xenografts
Source: Breast Cancer Res. 2020 Jun 23;22:68. doi: 10.1186/s13058-020-01300-y (PMC7310532; doi:10.1186/s13058-020-01300-y)
Supplement: Supplementary file 1 — Additional file : Supplemental Figure 1. IHC of PDX from which cell lines were derived. PDX were stained with antibodies to ER, PR, AR, CK5, and CK8/18. Slides were loaded into Aperio digital slide viewer and images captured at 40x magnification with Imagescope (Leica). Supplemental Figure 2. Nuclear-GFP labeled UCD cell lines for proliferation assays. Representative images of UCD cell lines during Incucyte analysis. Individual wells were captured at 6-18 h post plating. Mag bars,300 microns. Supplemental Fig. 3. Growth of UCD cell lines in vivo. One million cells were inoculated billaterally into the #4 mammary fat pads of NSG mice. Animals were supplemented with slow release estrogen pellets and tumors measure weekly. Mean plus SEM are indicated. N=6 tumors UCD12 and UCD47 and N = 3 tumors forUCD65. Supplemental Figure 4. Expressison profiles of UCD cell lines show moderate to strong positive correlation to their PDX tumors of origin. The RNA-seq expression profiles (FPKM) of UCD cell lines and UCD PDX were compared using the r package ggpubr. The r2 correlation and p values were plotted for each cell line and thecorresponding PDX. Supplementary Figure 5. Vimentin expression in ER negative UCD cell lines. UCD46, UCD115, and UCD178 cells were plated on glass slides in regular media. Cells were fixed and stained by immunocytochemistry with an antibody to vimentin with DAPI counterstain. Scale bar, 50 μM. [file 13058_2020_1300_MOESM1_ESM.pdf]

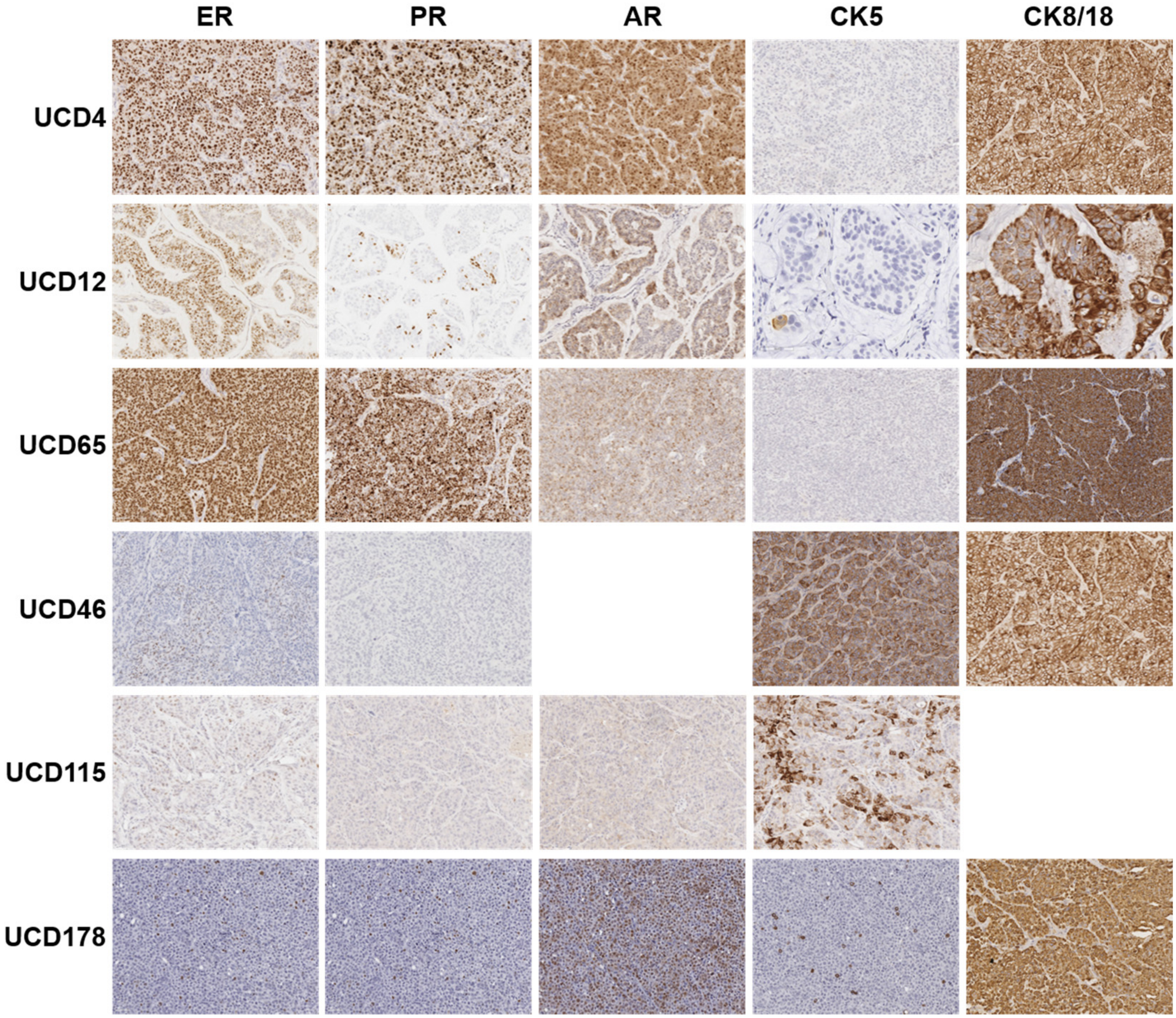

Supplemental Figure 1. IHC of PDX from which cell lines were derived. PDX were stained with antibodies to ER, PR, AR, CK5, and CK8/18. Slides were loaded into Aperio digital slide viewer and images captured at 40x magnification with Imagescope (Leica).

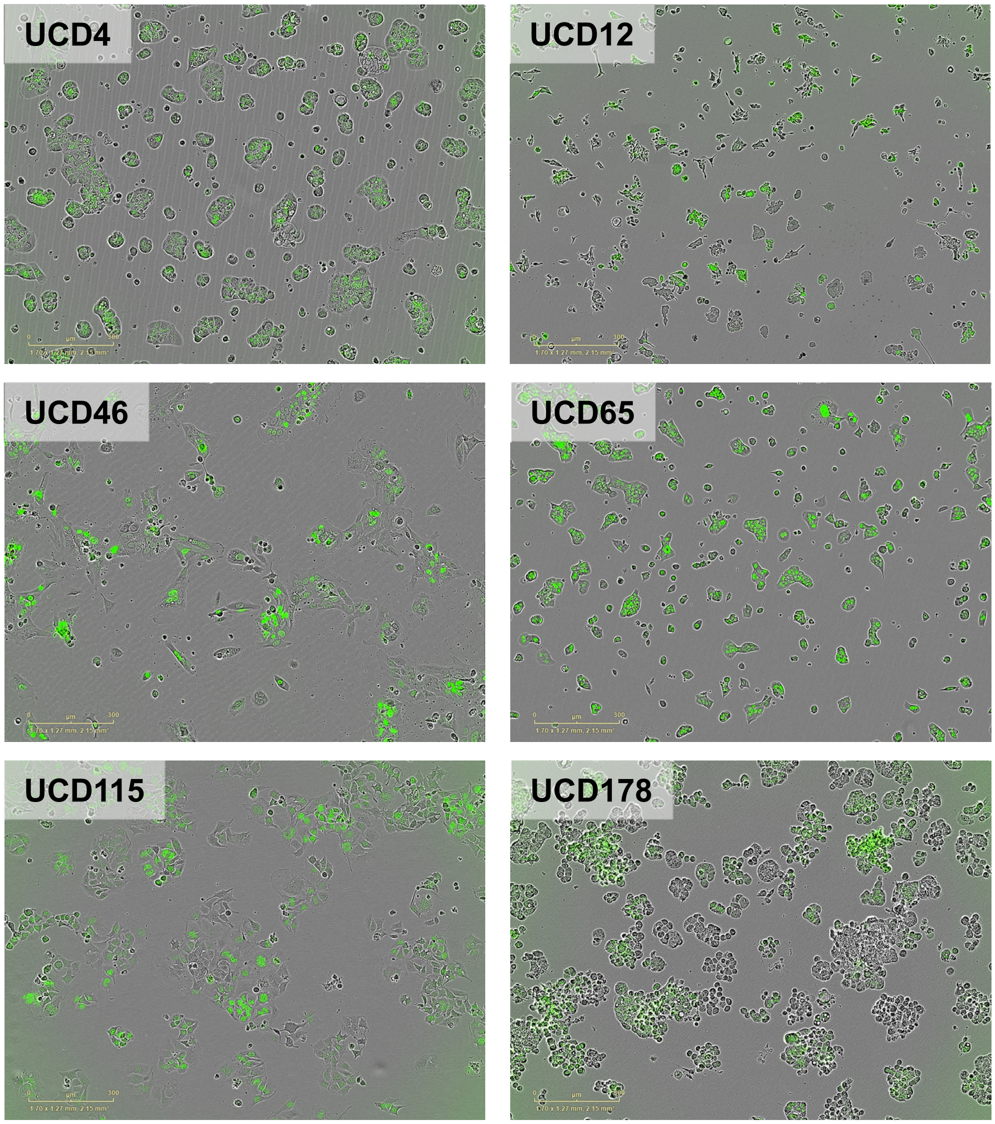

Supplemental Figure 2. Nuclear-GFP labeled UCD cell lines for proliferation assays. Representative images of UCD cell lines during Incucyte analysis. Individual wells were captured at 6-18 h post plating. Mag bars, 300 microns.

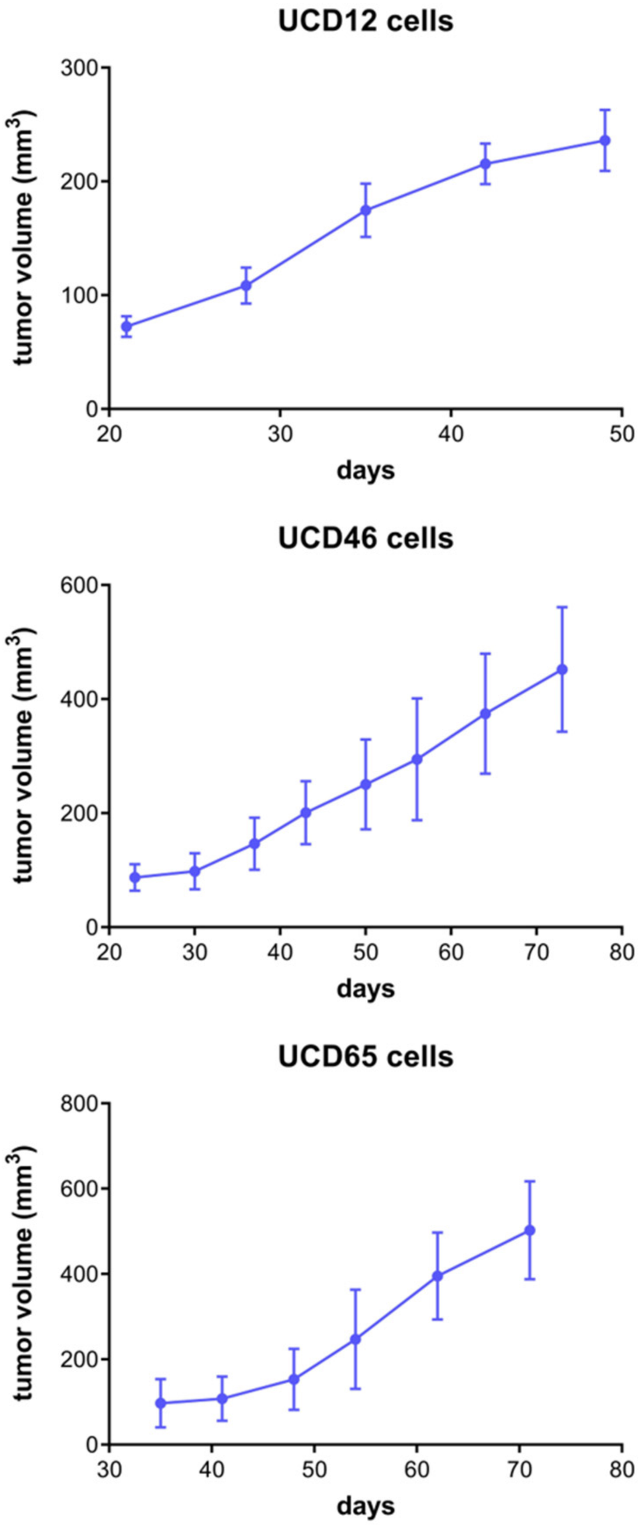

Supplemental Figure 3. Growth of UCD cell lines in vivo. One million cells were inoculated billaterally into the #4 mammary fat pads of NSG mice. Animals were supplemented with slow release estrogen pellets and tumors measure weekly. Mean plus SEM are indicated. N=6 tumors UCD12 and UCD47 and N=3 tumors for UCD65.

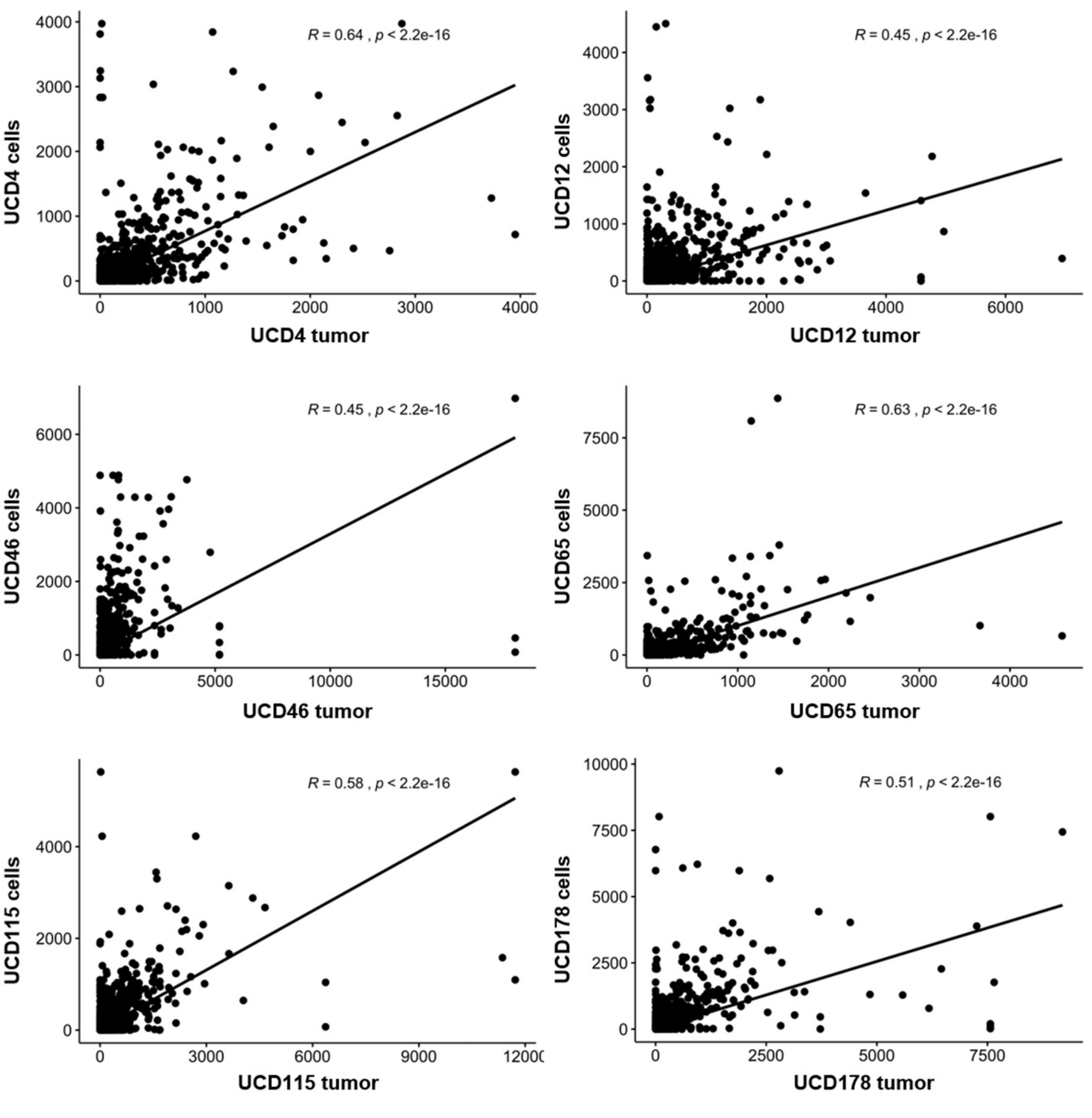

Supplemental Figure 4. Expressison profiles of UCD cell lines show moderate to strong positive correlation to their PDX tumors of origin. The RNA-seq expression profiles (FPKM) of UCD cell lines and UCD PDX were compared using the r package ggpubr. The r^2 correlation and p values were plotted for each cell line and the corresponding PDX.

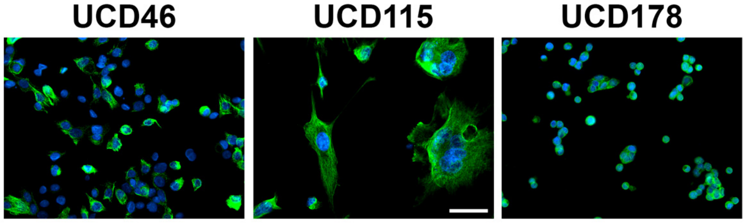

Supplementary Figure 5. Vimentin expression in ER negative UCD cell lines. UCD46, UCD115, and UCD178 cells were plated on glass slides in regular media. Cells were fixed and stained by immunocytochemistry with an antibody to vimentin with DAPI counterstain. Scale bar, 50 μM.
